# Office Visitors

**Published:** 1907-12

**Authors:** 


### OFFICE VISITORS.

We were notified by the office girl that a gentleman was in the reception room, when we entered a dignified, stately person arose to meet us, a man of three score and ten, at least, and perfect in manner and dress. "Do you know me Doctor?" I assured him as we always do, that his face was very familiar, but we could not recollect names, never could and many times came near forgetting our own. "Then you do not recognize one who forty years ago you saw every day, sometimes our meetings were pleasant, but sometimes we both wished we had not met and I am sorry to say that the unpleasant meetings were caused by you, for if not a bad boy you were very mischievous." We acknowledged that time had brought such changes in the physical appearance of each other, that a French teacher of our boyhood and his pupil, would not have known each other had they met on the street. As a handsome young graduate of a military school in France, he landed in the country just previous to the late war of the sixties, to escape military duty at home and took position in a popular private school and afterwards became one of the faculty of a University. His good looks, charming manners, ability, etc., secured for him a prominent place in society and he married rich. Now in his old age he was taking life easily in the city to which we had recently moved. "I heard you were here and I came to see if surely you were one of my old boys and also to have my teeth put in order and I am so glad to know that it is true, but I hope you will not take revenge on me for the punishments I gave you for not studying your French lessons and for your mischief as a boy."

We were exceedingly careful of him, operating for him most tenderly during the time of several sittings and in the intervals talked over the times and the people, of two score years past. "How the times have changed, the morale of the people is not the same, not the high tone among men, not the same delicate modesty and reserve among the ladies. legance and grace and politeness seem to have become accomplishments of the past and the mad rush after money and wealth and its consequent selfishness and roughness in the people of the present times prevail." The old gentleman was surely one of the old stock now rarely met in these practical times. He appreciated our humane operating and assured us in college parlance at the end of each sitting that he would give us a rating of "100". At the last sitting some little pain was given him from which he winced. "Take care MON AMI, you may not get that "100" today, no it shall be only "99". At the last when we had finished, we were told, "I like the way you have handled me and I shall not fear to visit you and the social features of a visit will not be marred by the usual disagreeableness of a professional service." We complimented him on the now purity of his English pronunciation, his physical preservation and devotion to his "old boys" and we parted for the time with mutual regard and respect. And the after thoughts of the reminiscences brought up by our meeting, have been a pleasure during our leisure moments since.

